# Symptom Burden, Medication Detriment, and Support for the Use of the 15D Health-Related Quality of Life Instrument in a Chronic Pain Clinic Population

**DOI:** 10.1155/2011/809071

**Published:** 2011-04-17

**Authors:** Bruce D. Dick, Saifudin Rashiq, Michelle J. Verrier, Arto Ohinmaa, Julie Zhang

**Affiliations:** ^1^Department of Anesthesiology and Pain Medicine, 8-120 Clinical Sciences Building, University of Alberta Edmonton, AB, Canada T6G 2B7; ^2^School of Public Health, 3-12 University Terrace, University of Alberta, Edmonton, AB, Canada T6G 2T4

## Abstract

Chronic noncancer pain is a prevalent problem associated with poor quality of life. While symptom burden is frequently mentioned in the literature and clinical settings, this research highlights the considerable negative impact of chronic pain on the individual. The 15D, a measure of health-related quality of life (HRQOL), is a user-friendly tool with good psychometric properties. Using a modified edmonton symptom assessment scale (ESAS), we examined whether demographics, medical history, and symptom burden reports from the ESAS would be related statistically to HRQOL measured with the 15D. Symptom burden, medication detriment scores, and number of medical comorbidities were significant negative predictors of 15D scores with ESAS symptom burden being the strongest predictor. Our findings highlight the tremendous symptom burden experienced in our sample. Our data suggest that heavier prescription medication treatment for chronic pain has the potential to negatively impact HRQOL. Much remains unknown regarding how to assess and improve HRQOL in this relatively heterogeneous clinical population.

## 1. Introduction

“Chronic noncancer pain” (CNCP) is a shorthand medical description for a constellation of symptoms in which individuals report pain for prolonged periods of time in the specific absence of incurable cancer. This distinction is made because the goals of and limitations upon symptom control in cancer versus noncancer pain are different. This pain may either be associated with a demonstrable, incurable structural illness or injury which is known to cause pain (such as arthritis or nerve damage), may continue long after the apparent structural resolution of such an illness or injury, or exist without any apparent structural explanation at all. Chronic noncancer pain conditions are very common in the industrialized world and are increasing in prevalence [1]. In some cases, it is associated with severe disability. Its direct and indirect costs to the sufferer and society as a whole are significant and well documented [2].

While objective physical correlates of pain exist, the experience of pain is entirely subjective [3]. The person's report of its presence and intensity is, therefore, definitive. Moreover, pain intensity fluctuates on a continuous basis and is also known to be affected by circumstance, thoughts, and feelings [4, 5]. The burden of symptoms experienced by chronic pain sufferers is frequently shared in clinical settings. These and other factors make pain intensity reduction a difficult outcome for therapists to use as a measure of the effectiveness of their care. Many clinicians place a high emphasis on observable improvements in health-related quality of life (HRQOL) as exemplars of successful treatment [6, 7]. 

Summary measures of HRQOL, whether obtained from structured interviews or, more commonly, questionnaires, are attractive to clinicians and researchers. They permit standardization of the data gathering process and render quantitative HRQOL measures that can be used to monitor individuals' progress with standard treatment, evaluate experimental treatment, or to compare HRQOL in populations with different characteristics. A plethora of HRQOL-measuring techniques are available [8], but there is no consensus on which tool has the best combination of reliability, validity, efficacy, and ease of use for the chronic pain population.

As a part of our Multidisciplinary Pain Centre's mandate to provide clinical care while extending the boundaries of the clinical research literature, we chose to explore HRQOL in our clinic population and compare it to data collected from another symptom scale previously adapted for use in our centre. In this paper, we describe the comparison of HRQOL measured by the 15D to the Edmonton symptom assessment scale [ESAS; 12]. The purpose of the present research was to determine whether there was a statistical relationship between HRQOL scores rendered by 15D and chronic noncancer pain—specific symptom burden measured by ESAS, with the specific null hypothesis that there would be no such relationship.

## 2. Materials and Methods

The Multidisciplinary Pain Centre at the University of Alberta Hospital in Edmonton, Alberta, is an out-patient referral facility for persons with chronic pain. The centre's patient population tends to deal with more complex and intractable pain problems than is often seen in most medical settings, accompanied by high degrees of medical comorbidity and mood disorders (e.g., depression and anxiety disorders). There is no charge to the patient for attending if he or she possesses valid health insurance from one of Canada's provincial health programs. 

The 15D [9, 10] is a preference-based, generic, self-administered measure of HRQOL. It encompasses 15 dimensions: mobility, vision, hearing, breathing, sleeping, eating, speech, elimination, the ability to perform usual activities, thinking and memory, discomfort and symptoms, depression, distress, vitality, and sexual activity. These factors are commonly measured in HRQOL instruments to capture a broad range of facets of life affected by health and particularly, poor health. Each dimension of the 15D is divided into 5 levels, from which the respondent selects the one which best describes his or her current state. Algorithms then employ a set of population-based preference or utility weights used to convert these scores into a utility, ranging from 0.00–1.00, respectively, indicating the value of death versus perfect health (no problems in any dimension). The 15D is easy to administer and respond to and has been demonstrated to have good test-retest reliability and discriminatory power that is comparable to other preference based instruments [10]. It is also available in the public domain without the somewhat prohibitive costs associated with other instruments. The 15D has been used as a measure of HRQOL in chronic pain populations before [11]. However, although intuitively suitable for this purpose, we have been unable to find any publications comparing the use of the 15D to other measures of HRQOL in this patient population. 

While a number of HRQOL measures have been used in CNCP studies, there is no gold-standard measure against which to compare 15D when seeking to quantify HRQOL in the chronic pain population. As a result, the opportunity to compare its discriminatory function to other pain-specific instruments has the potential to add meaningful information to the published literature. 

The ESAS [12] is a nine-item questionnaire, originally developed for the quantitative longitudinal assessment of distressing symptoms in patients receiving palliative care. The intensity of each of nine standard symptoms is reported on a 0–10 visual analogue scale, and there is the option for the respondent to add an additional tenth named symptom. These individual scores are then summed to give a total score. Our centre has adapted the ESAS for use in our CNCP population due to its ability to track symptom fluctuation in our chronic disease management setting. We adapted the ESAS originally to monitor symptom burden and patient progress in our centre years before we adopted the 15D as a standard outcome measure of HRQOL in our centre.

As part of their initial intake assessment, all patients completed the 15D. ESAS was also completed at intake and at each followup appointment. Patients completed the instruments with assistance from the clinic nurse. 

We retrospectively examined the records of 100 consecutive first attendees from February to October 2006. For each subject, we recorded age, gender, duration of pain, number of pain sites, and comorbid illnesses. We recorded the names and doses of each medication being used chronically, then quantified the total burden of each subject's medication using the medication quantification score III (MQS-III) [13], which assigns a measurement to each drug based on both the dose taken and its burdensomeness (derived from expert consensus). The 15D was recorded as described. ESAS at the intake visit was recorded using the following symptom categories: pain, tiredness, nausea, depression, anxiety, energy level, well-being, drowsiness, and constipation. Changes to the original published ESAS inventory were made in order to eliminate symptoms with limited relevance outside the palliative care context (lack of appetite and shortness of breath), to reword one symptom to make it more relevant for chronic pain patients (“activity”), and to include two others that are frequently cited by our population (tiredness and constipation). The optional tenth symptom was not added. Scores for “well-being” and “energy” were inverted prior to summing individual symptom scores to yield a total score with a potential range from 0–90.

Statistical analysis was performed using SAS version 9.2 for Windows. Descriptive statistics were obtained for the demographic variables and the two HRQOL measures. Using simple linear regression (PROC REG) with a backwards variable elimination algorithm, exploratory analysis of the relative effect of each of the other variables (age, gender, pain duration, number of pain sites, medication score, and number of comorbidities and ESAS) on 15D summary scores was performed in order to determine which variable(s) demonstrated the strongest independent correlation. The significance level for a variable to remain in the model at each stage was set at 0.05. To better illustrate the strength of significant associations, we then derived Pearson's rank correlation coefficient for the relationship between each of them and 15D. 

The Health Research Ethics Board of the University of Alberta approved this research with the consent of each participant.

## 3. Results

**Figure 1:**
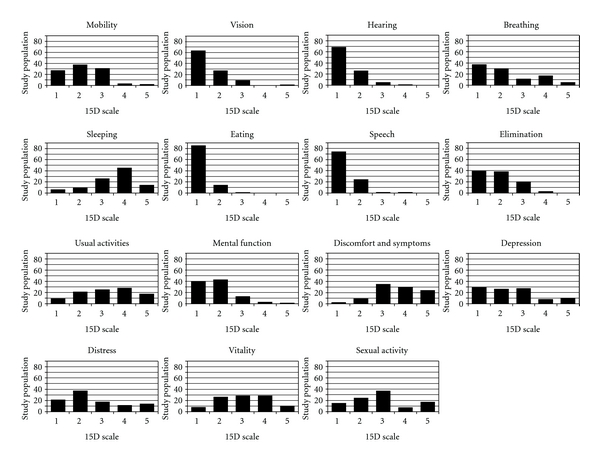
Number of responses to the component items of the ESAS.

The subject group is described in Tables 1, 2, and 3. The sample comprised 65% females. The typical subject was a person in middle age with a six-year pain history, one other medical or psychiatric comorbidity, and an MQS-III of 12, but pain duration, the number of pain sites, comorbidity count and MQS-III were all right skewed by a minority of subjects with much greater burdens in these areas. Fifty-eight percent of the subject group was consuming opioid analgesics. These ranged from short-acting low-potency opioids to high-dose long-acting preparations. Daily oral morphine equivalent doses ranged from 15 to 4800 mg/day (mean 292 mg, median 100 mg, IQR 143 mg). Other pain-related medications were also in common use, such as antidepressants (25%), NSAIDs (20%), sedative/hypnotics 15%, and anticonvulsants (11%).  Table 4 contains summary measures of MQS-III, ESAS and 15D. Graphical summaries of the responses to the component items of the 15D and ESAS are given in Figure 1. Correlations for the 15D, ESAS, and the number of comorbidities can be found in Table 5.

Of the dependent variables entered into the exploratory regression analysis, only ESAS, MQS, and number of comorbidities were retained as independent negative predictors of 15D (Table 4). ESAS showed the strongest association. The R^2^ for the model, (indicating the proportion of the total variance in 15D that was explicable by the predictors entered) was 0.31.

**Figure 2:**
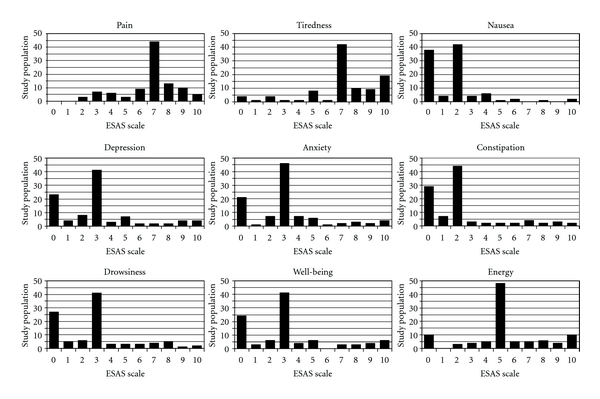
Number of responses to the component items of the 15D.

From a somewhat more qualitative standpoint, visual inspection of the histograms in Figures 1 and 2 highlights trends that are consistent with what is reported by individuals with chronic pain in Centers such as ours. The 15D/ESAS ratings of discomfort/pain, sleeping/tiredness, usual activities and well-being are all skewed toward patterns of experiencing increased problems and/or disability in these domains. There is clear convergence in these domains between questionnaires. As well, each of these areas is key to a person's global experience of quality of life. A somewhat unexpected trend was also noted that suggested a lower level of depression in this sample than is often reported in a center such as ours that deals with complex chronic pain [14]. It may be that antidepressant treatment in this population instituted prior to attending our clinic reduced the depression scores on the 15D.

## 4. Discussion

We have demonstrated inverse associations between HRQOL, as measured by the 15D instrument, and symptom burden in patients attending a referral treatment facility for chronic noncancer pain. This association is plausible and clinically relevant. Our null hypothesis is rejected. 

In attempting to determine the degree of colinearity between 15D and ESAS, our first requirement was that the study sample contains sufficient number of individuals who represented all degrees of symptom burden. In this respect, we are satisfied with the wide variation we recorded in both of these measures. The overall degree of symptom burden in our sample was high: The median ESAS score was 39 out of a possible 90. In contrast, a sample of medical oncology inpatients recorded median scores of 24.9 [15], while a series of patients receiving renal dialysis recorded mean scores equivalent to 34.1 [16]. Summary 15D scores are utilities, which is to say that a score 0.00 as represents a health state that is equivalent, in the respondent's mind to death, and 1.00 to perfect health. Using different methodology, Gold et al. [17] derived utilities for a large number of different medical states using data drawn from and validated on a large population sample of Americans, which enables us to contextualize our results. Our sample had a median utility of 0.66. In Gold's study of 131 named common conditions or disease states affecting all body systems, the lowest median utility was 0.21 (for hemiplegia) and the highest was 0.92 (for a number of conditions including acne, color blindness and hay fever). Nonpain conditions with median utilities equivalent to that reported by our chronic pain sample include retinal disease, absence of one hand or arm, breast cancer, and tachycardia.

One significant independent correlate of reduced HRQOL was the number of comorbidities reported by the subject. This is in keeping with recent work in which we derived independent risk factors for chronic pain in a large Canadian population sample [18]. This indicated that many chronic medical conditions are positively associated with chronic pain, even those, such as thyroid disease and COPD that are not conventionally thought of as being painful.

The high median MQS-III, indicating extensive use of prescription medication, is not surprising given the level of prescription medication use in Canada in general and for pain in particular [19]. Even so, this amount of consumption corresponds to the daily intake of the most potent opioid analgesic in the upper 50% of the therapeutic dose range [13]. Clinicians generally believe that by seeking to lower pain intensity with strong analgesics, the attenuation of social functioning associated with chronic pain will be reversed, primarily because pain is invoked as the reason for this decrease in functioning. Our results would seem to suggest that more intense drug treatment is not, in fact, associated with improved HRQOL. This may merely be a reflection of the fact that our sample was composed of first-time attendees to a referral centre, and thus it is biased towards those in whom drug treatment had not provided adequate pain control. An alternative interpretation, however, is that despite the salience of the link between pain intensity and decreased functioning, these are both simply consequences of a third, undescribed factor. Alternatively still, potent medications may be felt to be useful by patients and clinicians even though they worsen some aspects of HRQOL (because of side effects) or do not change it.

ESAS, MQS and the number of comorbidities between them only explained 31% of the variance in 15D that our sample reported. We know that HRQOL is a complex construct that reflects more than symptom burden. In Gold's study, for example, persons with ‘paraplegia' and those with “paralysis of the entire body” gave similar HRQOL scores, notwithstanding the fact that one would expect those with the continuing use of their arms to be less burdened. There are clearly subjective determinants of HRQOL that are not captured by the instruments we used. Most likely, there may also be important symptoms that are burdensome to chronic pain patients, but that were not captured in ESAS.

It may be worth noting that the distribution of ESAS scores visually appears to peak on some specific scores more than one might expect. There seems to be a disproportionate number of scores of 3 and 7 in our sample. While we have no data-driven hypotheses for this pattern, it is possible that this finding represents some kind of a response bias that requires further study when evaluating the use of the ESAS in this population.

## 5. Conclusion

Our findings suggest that the 15D is appropriate for use for individuals with chronic pain. As well, to the best of our knowledge, this is the first published study that has presented the use of the ESAS in a chronic pain population. While our methods do not formally validate its use statistically, our findings suggest that our modified ESAS has strong ecological validity and at least some degree of convergent validity when comparing some ESAS items to corresponding 15D items. Further study of how the ESAS could be beneficial for use with chronic pain populations is needed.

Our study includes a cross-sectional analysis of a complex hierarchy of determinants of health which are necessarily highly subjective in nature and rapidly changing. We have simply compared two different instruments, and we did not use a gold standard, since none exists. Nonetheless, our results suggest that the 15D instrument may be of use in the chronic pain clinic population to quantify general HRQOL as a utility measurement. This would permit cross-sectional comparisons between chronic pain and other conditions, the evaluation of therapeutic interventions over time, and the conduct of cost-effectiveness and cost-utility analyses in this clinically, socially, and economically important condition.

##  Funding

Departmental sources only.

##  Conflict of Interests

None, for any author.

## Figures and Tables

**Table 1:** Summary of participant characteristics.

Variable	Mean	Std Dev	Median	IQR	Minimum	Maximum
Age (yr)	48.7	15.8	49	19	17	92
Pain Duration (yr)	9.4	10.0	6	7	0.5	50
Number of Pain Sites	2.1	1.7	1	2	1	12
Number of comorbidities	1.0	1.3	1	2	0	6
MQS	13.3	11.0	12	12	0	42
ESAS	38	8	39	5	16	62
15D score	0.67	0.15	0.66	0.22	0.32	0.95

**Table 2:** Pain sites reported.

Site	% of sites reported (*n* = 213)
Back, hips, and buttocks	24
Lower extremity	23
Widespread	17
Chest	10
Head, face or jaw	8
Upper extremity	8
Neck	6
Abdomen, pelvis, and genitalia	6
Shoulder	5
Phantom	0.5

**Table 3:** Comorbidities by system (excluding 44 subjects reporting none).

Diagnostic category	Diagnosis	No. of patients	
Psychiatric	Depression	9	
	Anxiety	2	
	*All psychiatric*		*11*

CNS	Stroke	3	
	Migraine	1	
	Restless leg syndrome	1	
	Vertigo	1	
	Glaucoma	1	
	*All CNS*		*7*

Cardiovascular	Hypertension	11	
	Coronary disease	2	
	Valvular disease	2	
	*All cardiovascular*		*15*

Respiratory	COPD	2	
	Asthma	1	
	Sleep Apnea	1	
	*All respiratory*		*4*

Gastrointestinal	Reflux/dyspepsia	4	
	Inflammtory bowel disease	3	
	Irritable bowel syndrome	2	
	Fatty liver	1	
	Gallstones	1	
	Not specified	1	
	*All gastrointestinal*		*12*

Endocrine	Obesity	7	
	Hypercholesterolemia	4	
	Hypothyroidism	3	
	Diabetes	1	
	Osteoporosis	1	
	*All endocrine*		*16*

Renal	Chronic renal failure	2	
	Kidney transplant	1	
	*All renal*		*3*

Infectious	Hepatitis C	4	
	HIV	1	
	*All infectious*		*5*

Connective tissue/joint disorders	Osteoarthritis	3	
	Dematomyositis	1	
	Lupus	1	
	Vascultis	1	
	Congenital hip dislocation	1	
	*All connective tissue/joint*		*7*

Miscellaneous	Cancer in remission	3	
	Former street drug user	1	
	Data missing	1	
	*All miscellaneous*		*5*

**Table 4:** Independent predictors of 15D index from regression model.

Variable	Parameter estimate	Standard error	*F*	Pr > |*F*|
Intercept	0.9151	0.0745	150.77	<.0001
ESAS	−0.0074	0.0016	20.62	<.0001
MQS	−0.0033	0.0012	8.23	0.0051
Number of Comorbidities	−0.0230	0.0098	5.48	0.0213

*R* ^2^ = 0.31.

**Table 5:** Significant correlations with 15D index.

	ESAS	MQS	No. of comorbidities
r*	−0.42	−0.31	−0.22
*P*	<.0001	0.002	0.026

*Pearson's coefficient.
